# TNF-α protein synthesis inhibitor restores neuronal function and reverses cognitive deficits induced by chronic neuroinflammation

**DOI:** 10.1186/1742-2094-9-23

**Published:** 2012-01-25

**Authors:** Karim Belarbi, Timothy Jopson, David Tweedie, Carla Arellano, Weiming Luo, Nigel H Greig, Susanna Rosi

**Affiliations:** 1 Brain and Spinal Injury Center, University of California, San Francisco, California, USA; 2 Departments of Physical Therapy Rehabilitation Science and Neurological Surgery, University of California, San Francisco, California, USA; 3 Laboratory of Neurosciences, Intramural Research Program, National Institute on Aging, National Institutes of Health, Baltimore, Maryland, USA; 4 Brain and Spinal Injury Center, University of California San Francisco, San Francisco General Hospital, 1001 Potrero Ave, Bld#1, Room#101 94110, San Francisco, CA, USA

**Keywords:** Arc, Hippocampus, Immediate-early gene, Inflammation, Learning and memory, Tumor necrosis factor-α

## Abstract

**Background:**

Chronic neuroinflammation is a hallmark of several neurological disorders associated with cognitive loss. Activated microglia and secreted factors such as tumor necrosis factor (TNF)-α are key mediators of neuroinflammation and may contribute to neuronal dysfunction. Our study was aimed to evaluate the therapeutic potential of a novel analog of thalidomide, 3,6'-dithiothalidomide (DT), an agent with anti-TNF-α activity, in a model of chronic neuroinflammation.

**Methods:**

Lipopolysaccharide or artificial cerebrospinal fluid was infused into the fourth ventricle of three-month-old rats for 28 days. Starting on day 29, animals received daily intraperitoneal injections of DT (56 mg/kg/day) or vehicle for 14 days. Thereafter, cognitive function was assessed by novel object recognition, novel place recognition and Morris water maze, and animals were euthanized 25 min following water maze probe test evaluation.

**Results:**

Chronic LPS-infusion was characterized by increased gene expression of the proinflammatory cytokines TNF-α and IL-1β in the hippocampus. Treatment with DT normalized TNF-α levels back to control levels but not IL-1β. Treatment with DT attenuated the expression of TLR2, TLR4, IRAK1 and Hmgb1, all genes involved in the TLR-mediated signaling pathway associated with classical microglia activation. However DT did not impact the numbers of MHC Class II immunoreactive cells. Chronic neuroinflammation impaired novel place recognition, spatial learning and memory function; but it did not impact novel object recognition. Importantly, treatment with DT restored cognitive function in LPS-infused animals and normalized the fraction of hippocampal neurons expressing the plasticity-related immediate-early gene Arc.

**Conclusion:**

Our data demonstrate that the TNF-α synthesis inhibitor DT can significantly reverse hippocampus-dependent cognitive deficits induced by chronic neuroinflammation. These results suggest that TNF-α is a critical mediator of chronic neuroinflammation-induced neuronal dysfunction and cognitive impairment and targeting its synthesis could provide an effective therapeutic approach to several human neurodegenerative diseases.

## Background

Microglia activation is present during the early stages of many neurodegenerative conditions, such as Alzheimer's disease, Parkinson's disease and HIV-associated dementia [1-3]. Microglia are the resident innate immune cells of the central nervous system (CNS) and are critical in the early response and defense against insults that can impact the CNS. Microglia monitor their environment through a variety of pattern recognition receptors that respond to pathogen- or damage-associated molecular pattern stimuli, such as lipopolysaccharide (LPS) [4-6] and β-amyloid peptide [7-9]. Important as a first line of defense upon recognition of stressor stimuli, activated microglia accumulate at sites of tissue damage and express genes related to inflammation, such as proinflammatory cytokines and chemokines. Their over stimulation may, however, further activate inflammatory responses and thus potentiate a self-sustained cycle of unregulated inflammation [10]. Although microglia activation is required to guarantee CNS integrity, it has become clear that unregulated inflammatory responses and the release of proinflammatory molecules are responsible for neurotoxic processes in several neurodegenerative conditions. Laboratory data demonstrate that sustained microglia activation induced by LPS through the toll-like receptor (TLR)-4 mediated signaling pathway [5] results in an altered neuronal pattern of synaptic plasticity-related gene expression [11-14] and cognitive impairment in rodents [15,16].

Tumor necrosis factor (TNF)-α is a potent proinflammatory cytokine that plays a central role in initiating and sustaining the inflammatory response. TNF-α is generated as a transmembrane 26-kDa precursor molecule that is proteolytically cleaved to yield a soluble 17-kDa TNF-α protein that subsequently generates a non-covalently linked homotrimer in solution. Both membrane-bound and soluble forms of TNF-α are biologically active, signaling through two different transmembrane spanning receptor (R) subtypes, TNF-R1 and TNF-R2. TNF-α signaling has been shown to provide several important functions within the CNS [17] including regulation of microglial activation [18], regulation of glutamatergic transmission [19] and control of synaptic strength [20]. Elevated levels of TNF-α have been documented in several neurodegenerative disorders including Alzheimer's disease [21-24], Parkinson's disease [25,26] and HIV-associated dementia [27]. Hence, the regulation of TNF-α signaling may prove to be beneficial in the context of neurodegenerative disorders associated with neuroinflammation [28].

In accord with this, rapid clinical improvements in cognitive function have been described in subjects with Alzheimer's disease following intraspinal administration of the TNF-α-blocking agent etanercept, followed by Trendelenburg positioning [29-31]. Although a therapeutically relevant reduction of TNF-α signaling may be achieved by the use of such a protein based agent, its potential widespread utility in the broad context of neurological disorders may be limited consequent to its restricted passage across the blood-brain barrier. As an achievable alternative approach, the use of small lipophilic molecules possessing the ability to both inhibit TNF-α synthesis and penetrate the blood-brain barrier would be of significant value. 3,6'-dithiothalidomide (DT) is an isosteric analog of thalidomide (N-α-phthalimidoglutarimide), which has been reported to reduce the rate of TNF-α synthesis through enhanced degradation of its transcript [32,33]. DT possesses a TNF-α lowering action that proved more potent than equimolar thalidomide in both LPS-stimulated RAW 264.7 cells [34] and LPS-stimulated human peripheral blood mononuclear cells [35,36]. The *in vitro *TNF-α protein lowering properties of 3,6'-dithiothalidomide have been shown to translate into an *in vivo *setting where the agent ameliorated the increase in TNF-α protein levels induced by an acute peripheral LPS administration in rodent [37]. The present study was designed to investigate the therapeutic potential of DT in treating the consequences of chronic neuroinflammation using a relevant and well-established rat model displaying LPS-induced sustained microglia activation, and associated cognitive deficits [11-13,16,38,39].

## Methods

### 3,6'-dithiothalidomide

The DT compound was synthesized by WL according to a published procedure [40] to greater than 99.8% chemical purity. For dosing purposes, it was prepared as a suspension in 1% carboxymethyl cellulose to provide a final concentration of 56 mg/kg (equimolar to 50 mg/kg of thalidomide) and was administered by the intraperitoneal (i.p.) route (0.1 mL/100 g body weight injection).

### Animal procedures

This research was conducted under the supervision and with the approval of the University of California Institutional Animal Care and Use Committee. Twenty-eight 3-month-old male F344 rats (Charles River Labs) were used in this study. All rats were individually caged and maintained in a temperature- (22 ± 1°C) and light-controlled environment with an artificial 12 h light/dark cycle (lights off at 8 a.m. and on at 8 p.m.), with food and water freely available. As previously described, artificial cerebrospinal fluid (aCSF; n = 11) or lipopolysaccharide (LPS; n = 17; Sigma-Aldrich, E.coli, serotype 055:B5, TCA extraction, 1.0 μg/μl dissolved in aCSF) were loaded into an osmotic minipump (Alzet model #2004, 0.25 μl/h; 28-day delivery) and chronically infused for 28 days through a cannula implanted into the fourth ventricle of the brain [11-13,16]. At 29 days after surgery and the day following the end of chronic infusion, nine rats from the LPS-infused groups and five rats from the aCSF-infused group received DT via intraperitoneal injection for a duration of 14 days. In addition, eight rats from the LPS-infused groups and six rats from the aCSF-infused group were injected with 1% carboxymethyl cellulose alone. Rats were handled daily for 10 days prior to behavioral assessment. Behavioral manipulations and testing were carried out during the dark phase of the light cycle. Novel place and novel object recognition testing were performed from days 39 to 42 post-surgery and Morris water maze from days 44 to 48 post-surgery, as described in detail below. Each rat was euthanized by deep anesthesia followed by decapitation 25 min after completion of the probe trial of the Morris water maze and the brain was quickly removed and divided at the midline. One brain hemisphere was immediately frozen in -70°C isopentane (Sigma-Aldrich) for histological analysis and the hippocampus of other hemisphere was removed at 4°C and stored at -70°C for further RNA extraction. The same experimenter dissected all the samples for consistency.

### Novel place and novel object recognition test

An open field arena (60 × 60 cm square with 20 cm high walls) was placed in a dimly lit behavioral testing room. Large visual cues were placed on the walls of the testing room at various locations to provide spatial points of reference for the animals. Trials were recorded using an overhead camera and live tracking of the animals was achieved using an automated video tracking system (Ethovision XT; Noldus Information Technology). Exploratory behavior, defined as the animal directing its nose toward an object at a distance within a 4-cm radius, was analyzed during the first minute on each test unless specified. Data are expressed as the percent time spent exploring the target object ([time exploringtarget]/[time exploring target + time exploring non - target object] × 100); where the target object is represented by the relocated object during the place recognition task and by the novel object during the object recognition task. The arena and objects were cleaned with 0.025% bleach between trials to minimize odor cues.

### Novel place recognition

On assessment Days 1 and 2, rats were allowed to explore the open field arena for one 15 min period (habituation phase). On Day 3, two identical objects (A) were placed in opposite corners of the arena and secured to the floor using Velcro. Animals were allowed to explore the objects for 5 min (familiarization phase) and were then placed back in their cage for 5 min while one of the objects was moved to a new location within the arena (novel place/target object). Animals were then reintroduced into the open arena to explore for 5 min. The 24-h delay novel place/target object recognition test was conducted on day 4. The target object was moved to the remaining new location and the rats were allowed to explore for 5 min.

### Novel object recognition

For the novel object recognition task, two identical objects (B) were placed in the two corners/locations not used during the 24-h delay novel place recognition test. The animals were allowed to explore these objects for 5 min and returned to their cage for 5 min while one of the objects was replaced with a novel object (C, novel object/target object). Thereafter, animals were reintroduced into the box to explore for a further 5 min.

### Morris water maze test

A blue plastic tank/pool (160 cm diameter) was placed in a dimly lit behavioral testing room. Visual cues were placed around the room as spatial references. A transparent platform (12 cm diameter) was placed in the tank and water was added until the platform was submerged 1 cm below the surface. The test consisted of three phases: visible platform training, hidden platform training and a probe test.

Visible platform training took place on Day 1. A visible flag was installed on the escape platform, allowing the rat to visualize the location of the platform. Each rat performed a total of 4 visible platform trials, in which the rats learned to identify the visible platform when it was placed at different locations in each trial. A rat that failed to locate the platform within the allotted time was immediately guided to the platform manually. Once the rat reached the platform, it remained there for 20 s before removal from the water maze. Each rat was then towel-dried and returned to a heated incubator (25°C) to prevent hypothermia.

Hidden platform training took place on Days 2-4. Room and pool setup were identical to those of the visible platform training, with the exception that the flag was removed from the escape platform and the hidden platform was consistently located in the same location throughout all trials. All rats performed three training blocks per day (with two consecutive training trials per block) for 3 days (18 trials total), with the tank/pool insertion point location changing randomly from trial to trial. The maximum trial duration was 60 s, after which animals were manually guided to the platform in the event that they failed to locate it. Once animals reached the platform, they were allowed to remain there for 20 s. Animals were allowed a 60 s inter-trial interval within a given training block, and 60 min of recovery time between training blocks.

As a final assessment a probe trial was conducted on Day 5, 24 h after the last trial performed on Day 4. The platform was removed and animals were allowed to swim freely for 60 s. The percentage of time spent in each quadrant of the maze was then recorded. Specifically, an Ethovision automated video tracking system (Noldus Information Technology) monitored all performances in the following parameters: average velocity, escape latency and duration spent in each quadrant.

### RNA extraction and real-time RT-PCR analysis

The left hippocampus from each rat was processed for total RNA extraction and purification using the RNeasy Lipid Tissue Mini Kit (Qiagen). One microgram of total RNA was reverse-transcribed using the High-Capacity cDNA reverse transcription kit (Applied Biosystems). Quantitative real-time RT-PCR analysis was performed on Mx3005P QPCR System (Agilent Technologies) using Brilliant II SYBR Green reagents (Stratagene). The thermal cycler conditions were as follows: hold for 10 min at 95°C, followed by 40 cycles of a two-step PCR consisting of a 95°C step for 30 s followed by a 60°C step for 1 min. Primer sequences are presented in Table 1. Amplifications were carried out in triplicate and the relative expression of target genes was determined by the DDCT method, using cyclophilin gene expression as an internal control to normalize the results.

**Table 1:** Primer sequences

Gene	GenBank accession number	Forward primer 5'→3'	Reverse primer 5'→3'
**CD200**	AF231392	GTCCTTGGATGGGCATTTA	TGCGGAGATTCACCACAA
**Cyclophilin**	NM_017101	AGCATACAGGTCCTGGCATC	TTCACCTTCCCAAAGACCAC
**CX3CL1**	NM_134455.1	ATCCCAGTGACCTTGCTCATCC	AAGTGTCTGTGCTGTCTCGTCTCC
**Hmgb1**	NM_012963	GAGCACAAGAAGAAGCACCC	TAACGAGCCTTGTCAGCCTT
**IL-1β**	NM_031512	CACCTCTCAAGCAGAGCACAGA	ACGGGTTCCATGGTGAAGTC
**IL-4**	NM_201270	CAGGGTGCTTCGCAAATTTT	CTCAGTTCACCGAGAACCCC
**IL-6**	NM_012589	GCCAGAGTCATTCAGAGCAA	CATTGGAAGTTGGGGTAGGA
**IRAK1**	NM_001127555	ACCTCCCTGGAAGCTAGAGG	AGAGGCCAGGAACACTCTCA
**NfκB p65**	NM_199267	ACGATCTGTTTCCCCTCATCT	TGCTTCTCTCCCCAGGAATA
**TLR2**	NM_198769	TGGAGACTCTGGAAGCAGGT	CGCCTAAGAGCAGGATCAAC
**TLR4**	NM_019178	GAGGACTGGGTGAGAAACGA	AGATACACCAACGGCTCTG
**TNF-α**	NM_013693	GTGATCGGTCCCAACAAG	AGGGTCTGGGCCATGGAA
**TNFR1**	NM_013091	GCTGCACCAAGTGCCACAA	TCACACACCTCGCAGACTGTTTC
**TNFR2**	NM_130426	CACACATCCCTGTGTCCTTG	AAGCAGTTCGCCAGTCCTAA

### Histological procedures

The right brain hemispheres were blocked, as previously described [11,12,16], such that each block contained a half hemisphere from one rat from each experimental group. These blocks were then cryosectioned at a thickness of 20 μm and collected on microscopic slides. All slides were stored at -70°C until processed for immunohistochemistry. Three sections selected from the medial portion of the dorsal hippocampus (from 3.2 to 4.00 mm posterior to bregma) were stained for immunoreactive microglia using an antibody for major histocompatibility complex class II (MHC Class II RT1B mouse monoclonal antibody clone OX-6; BD Pharmingen) as previously described [11]. Six to eight additional slides from the same medial portion of the dorsal hippocampus were stained for Arc (activity-regulated cytoskeleton-associated protein) protein (rabbit polyclonal antibody; generously supplied by P.F. Worley's laboratory), as previously described [11].

### Image acquisition and analysis

Z-stack images (200× magnification; 1 μm optical thickness per plane; 8 planes) were taken using a Zeiss Apotome microscope and offline analyses were performed using Zeiss AxioVision software. The density of MHC class II immunoreactive cells and the percentage Arc immunoreactive neurons within the dentate gyrus (DG), and the CA1 and the CA3 areas of the hippocampus were then measured blindly to the experimental group, as described in detail previously [11,16].

### Statistics

Data are presented as means ± SEM. Differences between mean values were determined using *t*-test or one-way analysis of variance (ANOVA) procedures with Newman-Keuls tests for post hoc comparison. The learning curve of the Morris water maze hidden platform training was analyzed with a two-way ANOVA with day and experimental group as independent factors and with Newman-Keuls tests for post hoc comparison. Values of *p *< 0.05 are considered to be statistically significant. Data were analyzed and graphs were plotted by GraphPad Prism software version 5.0.

## Results

Chronic LPS infusion was well tolerated by all rats, in accord with previous studies [11]. Albeit that LPS-infused animals lost a few grams body weight immediately after surgery, all recovered within a few days and continued to gain weight normally for the duration of the study. Treatment with DT was well tolerated and, unlike thalidomide that is associated with soporific actions, did not impact spontaneous locomotion activity, open field exploration or other motor behaviors, such as swimming in the Morris water maze.

### Treatment with 3,6'-dithiothalidomide normalized TNF-α but not IL-1β levels

**Figure 1:**
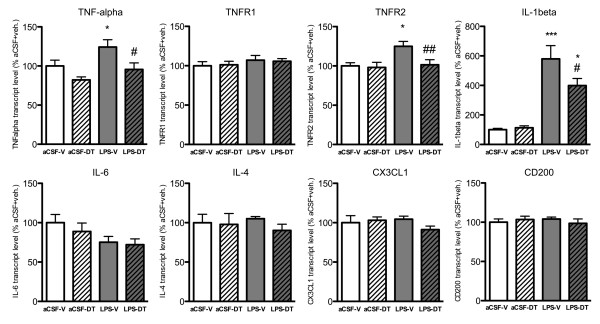
**Cytokine and chemokine gene expression**. TNF-α mRNA levels in the hippocampus were significantly elevated in LPS-vehicle rats (*p < 0.05 vs. aCSF-vehicle rats) and reduced to control levels in LPS-DT rats (#*p *< 0.05 vs. aCSF-vehicle rats). TNFR2 mRNA levels were significantly elevated in LPS-vehicle rats (*p < 0.05 vs. aCSF-vehicle) and similarly lowered to control levels in LPS-DT rats (##*p *< 0.01 vs. LPS-vehicle; not significant vs. aCSF-vehicle). IL-1β mRNA levels were significantly increased compared to aCSF-V levels in both LPS-vehicle and LPS-DT groups (***p < 0.001 and *p < 0.05 vs. aCSF-vehicle rats). However, DT treatment did induce a significant reduction in the gene transcript level compared to the LPS-vehicle group (#*p *< 0.05 vs. LPS-vehicle rats). Treatment with LPS and/or DT induced no significant differences in gene transcript levels for IL-6, IL-4 and CX3CL1 and CD200.

TNF-α expression in the hippocampus was assessed at the transcript level using real time PCR analysis (Figure 1). TNF-α mRNA levels were significantly elevated in LPS-vehicle rats as compared with aCSF-vehicle rats (129.97 ± 9.09%; *p *< 0.05 vs. aCSF-vehicle). Treatment with DT returned TNF-α mRNA to control levels (102.18 ± 8.90%; *p *< 0.05 vs. LPS-vehicle). Analysis of TNF-α receptors mRNA levels revealed that TNFR2 but not TNFR1 expression was significantly elevated in LPS-vehicle rats, as compared to aCSF-vehicle rats (124.91 ± 6.25%; *p *< 0.05 vs. aCSF-vehicle). Importantly, DT treatment decreased TNFR2 mRNA back to control levels (101.45 ± 6.46%; *p *< 0.01 vs. LPS-vehicle; not significant vs. aCSF-vehicle). IL-1β mRNA levels were significantly increased in both LPS-vehicle and LPS-DT rats, as compared with aCSF-vehicle rats (580.0 ± 89.36%; *p *< 0.001 and 399.3 ± 47.50%; *p *< 0.05 vs. aCSF-vehicle rats, respectively). However, treatment with DT in LPS-DT rats significantly attenuated IL-1β mRNA levels compared to the LPS-vehicle rats (*p *< 0.05). No significant differences were observed in the levels of the proinflammatory cytokine IL-6, the anti-inflammatory cytokine IL-4 or the chemokines CX3CL1 and CD200 for any treatment group (Figure 1). Together, our data demonstrate that treatment with DT significantly decreased LPS-induced elevations in gene transcripts for TNF-α and its receptor type 2 (TNFR2) and, to a lesser degree, IL-1β in the setting of chronic neuroinflammation. Interestingly, treatment with DT did not induce any significant changes in transcript measurements in the absence of an LPS-induced alteration in gene expression.

### Treatment with 3,6'-dithiothalidomide did not affect the numbers of MHC class II positive cells in the hippocampus

**Figure 2:**
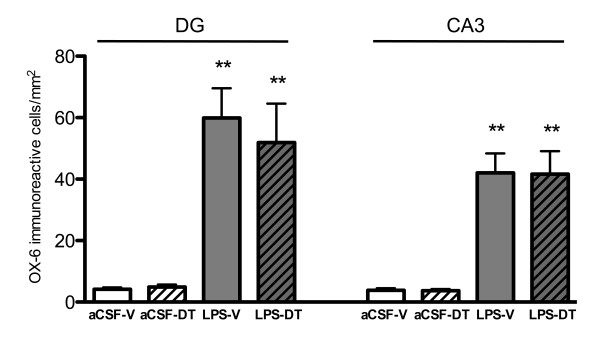
**OX-6 immunoreactive microglia staining**. Staining from the LPS-vehicle and LPS-DT rats displayed elevated numbers of OX-6 immunoreactive microglia per square millimeter within the DG and CA3 regions of the hippocampus (**p < 0.01 vs. aCSF-veh). No significant difference was observed between LPS-vehicle and LPS-DT rats.

Quantitative cell count analysis of OX-6 immunoreactive microglia revealed a significant increase of the number of MHC class II positive cells per square millimeter in the DG and CA3 areas of LPS-vehicle (59.91 ± 9.63 and 42.02 ± 6.37, respectively; *p *< 0.01 vs. aCSF-vehicle and aCSF-DT for both) and LPS-DT rats (51.81 ± 12.69 and 41.64 ± 7.46, respectively; *p *< 0.01 vs. aCSF-vehicle and aCSF-DT for both) (Figure 2). Importantly, the density of MHC Class II positive microglia did not differ between LPS-vehicle and LPS-DT in either the DG or the CA3. Very few MHC class II positive microglia were observed around the CA1 area of the hippocampus of LPS-vehicle (2.73 ± 1.01) and LPS-DT rats (2.41 ± 0.68) and no significant differences were observed across all four groups (data not shown). Our data show that DT treatment had no impact on altering the numbers of MHC class II positively stained microglia cells following their activation by chronic LPS infusion.

### Inhibition of TNF-α mRNA by 3,6-dithiothalidomide was paralleled by reduced expression of genes involved in TLR-mediated signaling pathways

**Figure 3:**
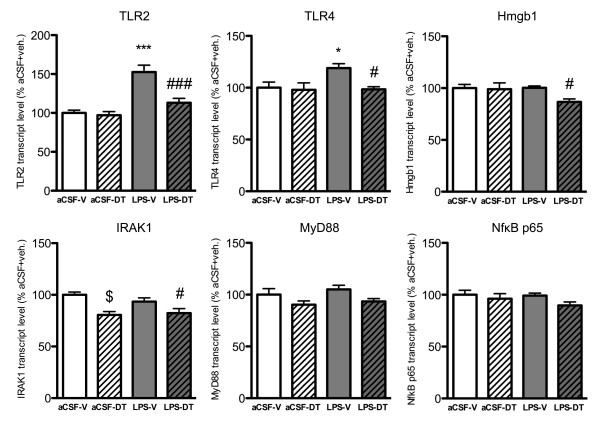
**TLR-mediated signaling pathway related gene expression**. Real time RT-PCR analysis from the hippocampus revealed that the gene expressions of TLR2 and TLR4 were increased in LPS-vehicle rats (***p < 0.001 and *p < 0.05 vs. aCSF-vehicle rats), while treatment with DT restored expression levels back to control (###*p *< 0.001 and #*p *< 0.05 vs. LPS-vehicle). Whereas LPS had no effect on Hmgb1 expression, treatment of LPS infused animals with DT decreased the expression of Hmgb1 (#*p *< 0.05 vs. LPS-vehicle). Treatment with DT reduced the expression of IRAK1 in both aCSF-DT and LPS-DT rats ($*p *> 0.05 vs. aCSF-vehicle and #*p *< 0.05 vs. LPS-vehicle). No significant differences in hippocampal transcript levels of MyD88 or NFκB p65 were observed between the treatment groups.

LPS is known to activate microglia primarily through the pattern recognition receptor TLR4 signaling pathway [4-6]. We therefore assessed the gene expression of factors involved in TLR-mediated signaling pathways which have been reported to be indicative of microglia activation following a central immune challenge [41,42] (Figure 3). TLR2 and TLR4 expression were increased in the hippocampus of LPS-vehicle rats (152.58 ± 8.89 and 118.95 ± 4.23%; *p *< 0.001 and *p *< 0.05 vs. aCSF-vehicle, respectively). Treatment with DT (i) normalized both TLR2 and TLR4 expression in LPS-DT rats (112.92 ± 5.71 and 98.47 ± 2.44%; *p *< 0.001 and *p *< 0.05 vs. LPS-vehicle, respectively; but not significant vs. aCSF-vehicle), (ii) decreased the expression of the endogenous TLR4 ligand Hmgb1 in LPS-DT rats compared with LPS-vehicle rats (86.63 ± 2.87 vs. 100.27 ± 1.74%; *p *< 0.05);, and (iii) reduced the expression of the enzyme, IRAK1, known to transduce signaling from TLRs in both aCSF-DT (80.59 ± 3.16 vs. 100.00 ± 2.73% in aCSF-vehicle; *p *< 0.05) and LPS-DT rats (82.35 ± 4.29 vs. 93.44 vs. ± 3.65% in LPS-vehicle; *p *< 0.05). Though MyD88 and NFκB p65 expression levels tended to be decreased in both aCSF-DT and LPS-DT, as compared with their respective control, statistical significance was not reached. Overall these data demonstrate that treatment with DT resulted in a lowered expression of several markers of the TLR-mediated signaling pathway.

### Treatment with 3,6'-dithiothalidomide rescued neuroinflammation-induced impairment in 5-min delay but not 24-h delay novel place recognition

#### Exploration during the novel place recognition familiarization phase

**Figure 4:**
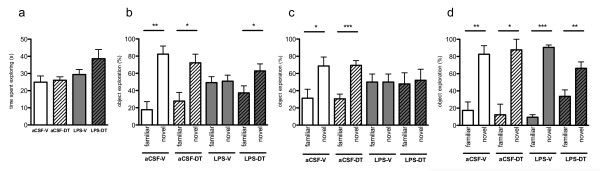
**Novel place and novel object recognition test**. (**A**). Novel place recognition familiarization phase. Analysis of the total amount of object exploration showed no significant difference across experimental groups. (**B**). 5 min delay novel place recognition. aCSF-vehicle, aCSF-DT and LPS-DT rats showed a preference for the novel place recognition over the familiar location (*p < 0.05 and **p < 0.01 vs. familiar place) whereas LPS-vehicle rats did not. (**C**). 24 h delay novel place recognition. aCSF-vehicle and aCSF-DT showed a preference for the novel place recognition over the familiar location (*p < 0.05 and ***p < 0.001 vs. familiar place) whereas LPS-vehicle and LPS-DT rats did not (**D**). Novel object recognition. aCSF-vehicle, aCSF-DT, LPS-vehicle and LPS-DT rats all showed a preference for the novel object over the familiar one (*p < 0.05; **p < 0.01; ***p < 0.001 vs. familiar object).

Analysis of the total time duration spent exploring in the open arena during the initial object-exploration familiarization phase revealed no significant difference between aCSF-vehicle (24.93 ± 3.64 s), aCSF-DT (26.03 ± 2.00s), LPS-vehicle (29.34 ± 2.97 s) and LPS-DT (38.60 ± 5.32 s) (*p *= 0.0965; Figure 4A).

#### Novel place object recognition test (5 min delay)

Animals from the aCSF-vehicle and aCSF-DT groups showed a preference for exploration of the object in the novel place over the familiar place (82.30 ± 9.39%; *p *= 0.0012 vs. familiar place; 72.21 ± 10.09%; *p *= 0.0144 vs. familiar place; respectively, Figure 4B). Interestingly, the LPS-vehicle group showed no overall preference to objects in either location (50.90 ± 6.98%; *p *= 0.86 vs. familiar place). Of relevance, the LPS-DT animals displayed a preference to the object placed in the novel location, which is more akin to the behavior of control animals (62.76 ± 8.18%; *p *= 0.0445 vs. familiar place). These data suggest that DT treatment rescued neuroinflammation-induced impairment in the novel place object recognition assessment after a 5-min delay interval. Analysis of the cumulated exploration duration (novel + familiar location) showed no significant differences between groups (data not shown).

#### Novel place object recognition test (24 h delay)

Similar to that observed in the 5 min delay interval groups, aCSF-vehicle and aCSF-DT animals displayed a preference for the novel object place rather than the familiar object place (68.70 ± 10.39%; *p *= 0.0216; 69.43 ± 5.49%; *p *= 0.0005; respectively) (Figure 4C). However, in contrast to the 5 min delay interval, both the LPS-vehicle and LPS-DT groups showed no preference for the novel object place over the familiar object location (50.02 ± 9.32%; *p *= 0.9981; 52.09 ± 12.90%; *p *= 8219; respectively, Figure 4C). Analysis of the cumulated exploration duration (novel + familiar location) showed no significant differences between groups (data not shown).

### Novel object recognition test was not affected by chronic neuroinflammation

#### Exploration during the novel object recognition familiarization phase

Analysis of the total time spent in exploration of the objects in the open arena during the novel object recognition familiarization phase revealed no significant difference between groups (data not shown).

#### Novel object recognition test (5 min delay)

Short-term novel object recognition was used to assess hippocampus-independent memory [43,44]. All animals across treatment groups showed a significant discrimination between the novel and familiar objects, spending a significantly higher percentage of time exploring the novel object over the familiar one (aCSF-vehicle: 82.65 ± 9.89; *p *= 0.0016; aCSF-DT: 87.67 ± 12.33; *p *= 0.0124; LPS-vehicle: 90.52 ± 2.89; *p *< 0.0001; LPS-DT: 66.28 ± 7.46; *p *= 0.0094 vs. familiar place), thereby indicating that neither neuroinflammation nor treatment with DT altered novel object recognition (Figure 4D).

### Treatment with 3,6'-dithiothalidomide restored acquisition and consolidation of spatial memory

#### Visible platform training

**Figure 5:**
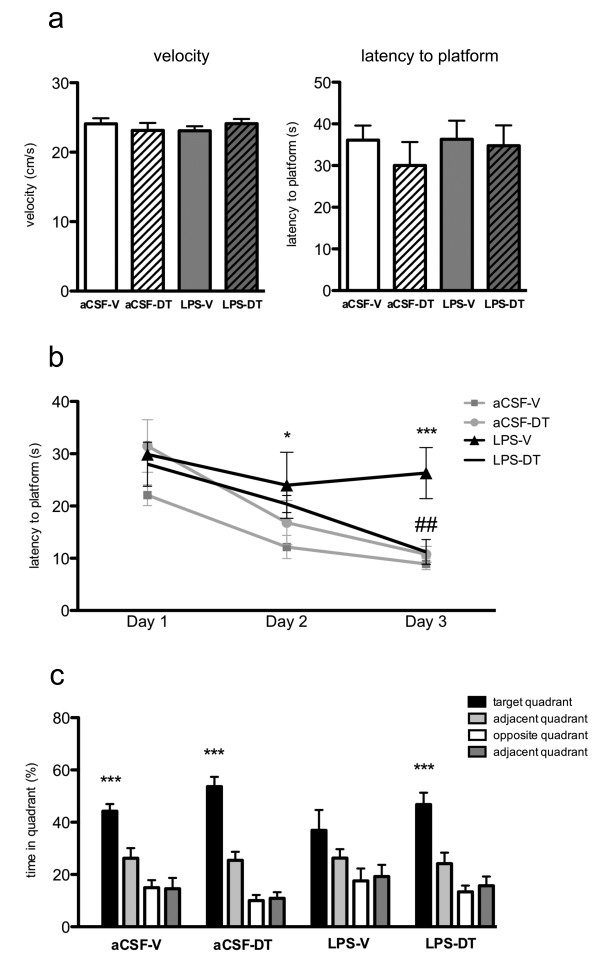
**Morris water maze test**. (**A**). Visible platform training. There was no significant group difference in average swim velocity or escape latency to the platform (**B**). Hidden platform training. aCSF-vehicle, aCSF-DT and LPS-DT groups improved their daily performance during the acquisition phase of the Morris water maze task, whereas LPS-vehicle rats showed an impaired learning profile. The average escape latency of LPS-vehicle rats was significantly higher than that of aCSF-vehicle animals on the second (*p < 0.05) and third day (***p < 0.001). The average escape latency of LPS-DT rats did not significantly differ from aCSF-vehicle rats, and was significantly lower than that of LPS-vehicle animals on the third day of training (^##^*p *< 0.01). (**C**). Probe test. aCSF-vehicle, aCSF-DT and LPS-DT groups spent a significantly higher percentage of time in the target quadrant than the other quadrants (***p < 0.001 vs. other quadrants) whereas LPS-vehicle rats did not.

All treatment groups (aCSF-vehicle, aCSF-DT, LPS-vehicle and LPS-DT) displayed a similar swim velocity (24.08 ± 0.81; 23.15 ± 1.07; 23.09 ± 0.64 and 24.10 ± 0.69 s/m, respectively; *p *= 0.6878) and similar escape latency to locate the platform (36.10 ± 3.51; 30.00 ± 5.62; 36.33 ± 4.44 and 34.78 ± 4.86 s, respectively; *p *= 0.7775), suggesting that neither LPS or DT treatment induced any impairments in motor function or visual acuity to find the platform (Figure 5A).

#### Hidden platform training

Interestingly, animals form the aCSF-vehicle, aCSF-DT and LPS-DT groups showed daily improvements in their abilities to locate the hidden platform during the acquisition phase of the Morris water maze task, whereas LPS-vehicle rats showed an impaired learning profile (Figure 5B). This delayed learning of LPS-vehicle versus aCSF-vehicle rats, was significant on the second (*p *< 0.05) and third days (*p *< 0.001) of the hidden platform training. The improved learning of LPS-DT rats, compared with LPS-vehicle rats was significant on the third day of training (*p *< 0.01). These disparities were not caused by differences in swim speed (data not shown).

#### Probe test

Findings from the probe test indicate that animals from the aCSF-vehicle, aCSF-DT and LPS-DT groups spent a significantly higher percentage of time in the target quadrant (the location that contained the platform during training) as opposed to the other equivalent zones (44.22 ± 2.78%; 53.63 ± 3.74; 46.79 ± 4.52%; *p *< 0.0001 vs. other quadrants for both). In contrast, the LPS-vehicle group failed to show significant discrimination between any quadrant (*p *= 0.0932, Figure 5C).

### Treatment with 3,6'-dithiothalidomide normalized the expression of the plasticity-related arc in the hippocampus

**Figure 6:**
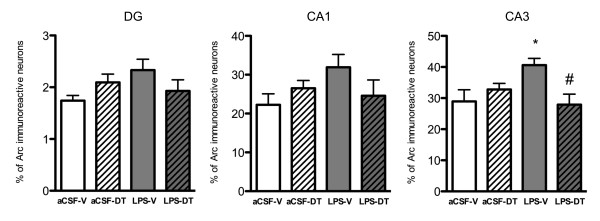
**Proportion of neurons expressing Arc in response to behavior**. The proportion of pyramidal neurons expressing behaviorally-induced Arc in the CA3 area was significantly increased in LPS-vehicle animals (*p < 0.05 vs. aCSF-vehicle). DT normalized this proportion back to control levels in LPS-DT rats (#*p *< 0.05 vs. LPS-vehicle). Similar trends were observed in the DG and the CA1 area, albeit statistical significance was not achieved.

To establish how neuroinflammation affected cognitive information processing and the subsequent effects of treatment with DT, we analyzed the proportion of neurons expressing the plasticity-related immediate early gene Arc after the 24 h probe test trial in the DG, CA1 and CA3 areas of the hippocampus (Figure 6). The percent of neurons expressing Arc within the LPS-vehicle group showed an elevated trend, as compared to the aCSF-vehicle group, in all the areas analyzed that reached statistical significance in the CA3 area (40.59 ± 3.74 in LPS-vehicle vs. 28.95 ± 3.74% in aCSF-vehicle; *p *< 0.05). In LPS-DT rats, the percent of neurons expressing behaviorally-induced Arc protein within the DG, the CA1 and the CA3 was lower than that observed in LPS-vehicle animals, with statistical significance similarly observed in the CA3 (27.89 ± 3.37%; *p *< 0.05 vs. LPS-vehicle).

## Discussion

Chronic neuroinflammation is consistently present during the early stages of Alzheimer's disease and HIV-associated dementia and likely contributes to the cognitive dysfunction associated with these disorders and drives disease progression [1,45-47]. It has hence become important to understand the mechanisms underlying chronic neuroinflammation-induced cognitive impairments and to explore potential therapeutic approaches to ameliorate them. Chronic neuroinflammation can be reliably reproduced in rodents by the slow, continuous infusion of a low dose of LPS into the brain, and such models are associated with impaired spatial cognitive function [11,15,16]. Herein we were able to demonstrate that the blood-brain barrier permeant TNF-α lowering agent, DT, effectively rescued hippocampus-dependent cognitive impairment induced by chronic neuroinflammation. The decrease in TNF-α mRNA was paralleled with a normalization of the inflammation-induced disproportionate expression of the plasticity-related immediate-early-gene Arc. These data suggest that the proinflammatory cytokine TNF-α signaling is critically involved in the disruption of patterns of hippocampal activity underlying learning and memory and that inhibition of TNF-α synthesis can restore cognitive function from a behavioral and cellular prospective.

Chronic neuroinflammation is characterized by a long-standing activation of immune cells and subsequent sustained release of proinflammatory factors. Here chronic neuroinflammation was induced by slow intraventricular infusion of LPS, a component of the outer membrane of Gram-negative bacteria, known to selectively activate microglial cells through the stimulation of TLR4/CD14 receptors [4-6]. This treatment gave rise to a long-lasting innate immune response that persisted in the hippocampus even following discontinuation of LPS infusion, as evidenced by increased gene expression of the inflammatory markers TNF-α, IL-1β, TLR2 and TLR4. DT was administered after discontinuation of LPS infusion to define the ability of the compound to reverse neuronal dysfunction and cognitive impairments induced by self-sustained chronic neuroinflammation.

Moreira and co-workers described that thalidomide reduced the half-life of TNF-α mRNA by approximately 50% [32]. Latterly, Zhu and Greig and co-workers illustrated that thiol analogs of thalidomide, including DT, were more potent than thalidomide at lowering LPS stimulated TNF-α protein secretion *in vitro*. Additionally, the thiol analogs were found to share the same mechanism of action to that of thalidomide where the agents acted upon the 3'-UTR of TNF-α mRNA [35,36]. Interestingly, several protein mRNA's are subject to regulation at the 3'-UTR region via actions upon adenylate/uridylate (AU)-rich elements. TNF-α mRNA is known to be regulated by several RNA binding proteins such as HuR which stabilizes RNA and effectively increase the translation efficiency of the cytokine [48], tristetraprolin and AUF1 both of which destabilize RNA and thus reduce the translation of RNA into protein [49,50]. Both HuR and tristetraprolin are regulated by p38 mitogen-activated protein kinase (p38 MAPK) and p38 MAPK has been shown to be inhibited by thalidomide [51,52]. It is likely that DT acts in a similar manner, however additional studies will be required to establish this.

The chosen dose and duration of DT administration were based on preliminary results showing that 14 days of DT administration (56 mg/kg/day; i.p.) reduced microglia activation and prevented cognitive impairments induced by concomitant LPS infusion (Tweedie D, Rosi S, Greig NH, unpublished work). The dose of DT (56 mg/kg, equimolar to 50 mg/kg of thalidomide) compares favorably with those of thalidomide used in humans, where doses of up to 1200 mg are administered. Hippocampal gene expression analysis showed a normalization of TNF-α levels in LPS-infused rats treated with DT and confirmed the compound's ability to access the CNS and to act as a TNF-α synthesis inhibitor, in line with previous studies [34-37]. The increase in TNF-α levels and its normalization by DT were paralleled by similar modulation of TNFR2. TNFR2 is known to be expressed primarily by cells of the immune system (including microglia) and by endothelial cells and to act via a number of different signaling pathways to increase NFκB-mediated transcription of anti-apoptotic and pro-inflammatory gene targets [17,53]. The normalization of TNF-α by DT was not associated with major variations of other cytokines and chemokines known to regulate inflammation (IL-1β, IL-6, IL-4, CD200 and CX3CL1). Importantly, IL-1β levels remained significantly elevated, suggesting that DT effects were mediated primarily through TNF-α but not IL-1β mediated signaling pathway inhibition.

TNF-α has been shown to promote the proliferation and activation of microglia cells [17,18]. Therefore, we investigated whether microglia activation could be modified by DT treatment, after the initiation and onset of microglial activation. We observed that DT treatment did not impact the number of MHC-Class II positive microglia accumulated within the DG and CA3 regions of the hippocampus in response to prior LPS-infusion. The elevated expression of genes involved in the TLR-mediated signaling pathways has been reported to be indicative of classical microglia activation following a central immune challenge [41,42]; in line with these findings, our data showed increased expression of TLR2 and TLR4 levels in LPS-vehicle rats. Importantly, normalization of TNF-α transcripts by DT was paralleled by a significant decline in the expression of several genes involved within the TLR mediated signaling pathways, such as TLR2, TLR4, Hmgb1 and IRAK1. As it is established that TLR-mediated signaling pathway activation leads to increased TNF-α expression [54], we can hypothesize that this, in turn, contributed to the decrease in TNF-α expression observed in our experiment. As MHC class II expression can also occur in microglia that are alternatively activated to produce anti-inflammatory cytokines, we also studied the expression of the anti-inflammatory cytokine IL-4; however no differences were observed across treatment groups. Our findings suggest that the decrease of TNF-α led to an attenuated expression of genes involved within the TLR-mediated signaling pathway associated with classical microglia activation.

Rats with chronic neuroinflammation displayed impaired performances in both novel place recognition and spatial learning and memory retention tests, but not in the novel object recognition test. The processing of spatial recognition memory and the ability to remember where an event occurs within an allocentric place have been shown to be highly dependent on the hippocampus [43,55,56]. In contrast, findings from several lesion and imaging studies suggest that the ability to recognize an object that was part of a previous recent event is not hippocampus dependent but relies on the perirhinal cortex [43,55,57,58]. In this context, our data therefore indicate that chronic neuroinflammation impaired hippocampus-dependent but not hippocampus-independent cognition. This behavioral phenotype is highly consistent with the fact that neuroinflammation is primarily distributed within the hippocampus in our model [11,15] and further demonstrates the high vulnerability of the hippocampal formation toward neuroinflammation, as is also the case in humans. Importantly, the cognitive deficits observed in this study were observed up to 3 weeks after LPS infusion ended, which is in accord with our recent study showing that neuroinflammation persists for at least 2 months following termination of LPS-infusion [13]. Taken together, our data suggests that, once initiated, neuroinflammation time-dependently persists, significantly impacts neuronal function and leads to hippocampus-dependent cognitive deficits.

Importantly, chronic neuroinflammation resulted in both short- and long-term spatial recognition memory deficits as measured following 5-min and 24-h delays. Short-term memory relies on synaptic mechanisms that do not require protein synthesis and is insensitive to inhibitors of transcription and translation. In contrast, storage of long-term memory, or memory consolidation, requires protein synthesis-dependent changes in synaptic strength [59-63] and the expression of the plasticity related immediate early gene Arc [64,65]. Thus, our data support the concept that both protein synthesis-independent and protein synthesis-dependent forms of synaptic plasticity were altered by chronic neuroinflammation. In addition to its well-known role in inflammation [17], TNF-α has direct effects on glutamate transmission and, notably, has been shown to increase AMPA receptor surface expression [20,66]. The trafficking of AMPA receptors is thought to underlie, at least in part, both the rapid form of synaptic plasticity and long-lasting and protein synthesis-dependent changes in synaptic strength [67]. In line with previous reports [68,69], our findings suggest that prolonged induction of TNF-α during chronic neuroinflammation may contribute to a dysregulation of synaptic homeostasis causing short-term recognition and long-term spatial memory deficits.

Inhibition of TNF-α synthesis by DT was able to restore the 5 min delay spatial recognition memory, indicating that normalization of TNF-α levels was associated with a significant amelioration of synaptic homeostasis underlying short-term memory. In contrast, normalization of TNF-α was not sufficient to restore the 24 h delay spatial recognition. The difference in the ability of DT to rescue the 5 min but not 24 h delay recognition memory likely was not due to variations in its bioavailability, because the DT intraperitoneal injections were performed daily at the same time during the novel place recognition protocol and all tests were conducted at similar hours every day. Interestingly, inhibition of TNF-α by DT was able to restore both spatial learning and 24 h retention probe trial performance in the Morris water maze. The apparent discrepancy between the rescue by DT of long-term spatial memory in the Morris water maze but not in the novel place recognition may be due to differences between these tasks, such as the nature of the motivation or the amount of training performed. Indeed, although both assess long-term spatial memory, the Morris water maze performance relies on escape behavior, speed and accuracy of spatial navigation whereas novel place recognition relies on a novelty spontaneous preference paradigm. The amount of training also significantly differs between these tasks and was more important for the Morris water maze than for novel place recognition (see methods). We have recently demonstrated that additional training in the water maze paradigm can, in fact, mitigate initial mild cognitive deficits [70]. Finally, the Morris water maze task was conducted subsequent to the novel place recognition test and rats possibly gained ability to process spatial information through their behavioral training. Thus, any of these variables could potentially interact with the consequences of DT treatment in ways that a beneficial effect was evidenced in the 24 h delay probe trial of the Morris water maze probe trial but not in the 24 h delay novel place recognition.

The plasticity-related immediate-early gene *Arc *and its protein product are induced in hippocampal neurons following spatial exploration in percentages similar to those recorded electrophysiologically [12,71,72]. *Arc *is involved in the trafficking of AMPA glutamate receptors [73] and Arc protein is required in the engagement of durable plasticity processes that underlie memory consolidation [64,65]. In the present study, we used the detection of Arc as a reliable method for monitoring cellular activity reflecting spatial and contextual information processing in response to behavior (i.e. the Morris water maze probe trial) [12,14,71]. We previously demonstrated that chronic neuroinflammation disrupts the expression pattern of behaviorally-induced Arc in the DG and CA3 after novel environment exploration [11,12]. Both the DG and CA3 hippocampal subregions cooperate to efficiently process spatial information [74]. The CA3 area is thought to be particularly involved in large scale spatial representation, and lesions of the CA3 causes extensive spatial processing difficulties [74,75]. Here, we report that the percentage of pyramidal neurons expressing Arc in the CA3 area was significantly dysregulated in LPS-vehicle rats with impaired memory performance. Importantly, the beneficial effect of DT on cognitive function was paralleled by a normalization of Arc *de novo *protein synthesis expression. As Arc transcription is regulated by AMPA receptors [76], we can hypothesize that the prolonged TNF-α increase contributes to the disruption in expression of behaviorally-induced Arc through a dysregulation of glutamate signaling homeostasis. It is thus conceivable that the reduction of TNF-α levels by DT did restore ideal Arc expression levels necessary to maintain optimal synaptic plasticity. The close correlation between Arc expression levels and probe trial performance across all experimental groups further strengthens the significance of Arc as a reliable molecular marker of neuronal plasticity related to memory.

## Conclusions

The present study demonstrates that inhibition of TNF-α synthesis by 3,6'-dithiothalidomide reverses hippocampus-dependent cognitive deficits induced by chronic neuroinflammation. To our knowledge this is the first time that such an effect was reported. Taken together, our data support that TNF-α is a critical mediator of chronic neuroinflammation-induced neuronal dysfunction and cognitive impairment. Therefore, pharmacological strategies aimed at decreasing TNF-α synthesis may be useful to restore neuronal functions and consequently reduce cognitive deficits during chronic neuroinflammation. All together these data suggest that selectively targeting the TNF-α pathway using a blood brain barrier-permeable compound could provide an effective therapeutic approach to several human neurodegenerative diseases.

## Abbreviations

(DT): 3,6'-dithiothalidomide; (ANOVA): Analysis of variance; (Arc): Activity-regulated cytoskeleton-associated protein; (aCSF): Artificial cerebrospinal fluid; (CNS): Central nervous system; (LPS): Lipopolysaccharide; (MHC Class II): Major histocompatibility complex class II; (TLR): Toll-like receptor; (TNF): Tumor necrosis factor.

## Competing interests

The authors declare that they have no competing interests.

## Authors' contributions

KB contributed to the design of the study, performed transcript, behavioral and histological analyses, analyzed the experiments, wrote the manuscript; TJ and CA performed behavioral and histological analyses; WL synthesized the DT compound; DT and NG contributed to the design of the study and revised the manuscript; SR designed the experiment, performed the surgeries, analyzed the experiments and wrote the manuscript. All authors read and approved the final manuscript.
